# Family-based intervention to prevent childhood obesity among school-age children of low socioeconomic status: study protocol of the FIVALIN project

**DOI:** 10.1186/s12887-021-02697-x

**Published:** 2021-05-21

**Authors:** C. Homs, P. Berruezo, G. Segn, L. Estrada, J. de Bont, J. Riera-Roman, E. Carrillo-lvarez, H. Schrder, R. Mil, S. F. Gmez

**Affiliations:** 1 Gasol Foundation, Sant Boi de Llobregat, Spain; 2 Faculty of Psychology, PSITIC Research Group, Education and Sport Sciences Blanquerna Universitat Ramon Llull, Cster, 34 08032 Barcelona, Spain; 3 Gasol Foundation, Los Angeles, CA USA; 4 ISGlobal, Barcelona, Spain; 5 CIBER Epidemiology and Public Health (CIBERESP), Carlos III Health Institute, Madrid, Spain; 6 Universitat Pompeu Fabra, Barcelona, Spain; 7 Fundaci Institut Universitari per a la recerca a lAtenci Primria de Salut Jordi Gol i Gurina (IDIAPJGol), Barcelona, Spain; 8 Universitat Autnoma de Barcelona, Cerdanyola del Valls, Spain; 9 Global Research on Wellbeing (GRoW) research group, Blanquerna School of Health Sciences Universitat Ramon Llull, Padilla, 326-332 08025 Barcelona, Spain; 10 Cardiovascular Risk and Nutrition Research Group (CARIN), IMIM (Hospital del Mar Medical Research Institute), Barcelona, Spain; 11 GREpS, Health Education Research Group, Nursing and Physiotherapy Department, University of Lleida, Lleida, Spain

**Keywords:** Pediatric obesity, Primary prevention, Low-income population, Health status disparities, Healthy lifestyle

## Abstract

**Background:**

Childhood obesity represents a global public health crisis: the number of obese children and adolescents (aged 519years) worldwide has risen tenfold in the past four decades. The vast majority of overweight and obese children live in high-income countries, and low socio-economic status (SES) is a significant risk factor. Family Based Interventions (FBI) have demonstrated positive results in preventing obesity, although these results are strongly influenced by SES. Moreover, we still poorly understand how FBI can determine a positive trend in weight status in low-income communities. Therefore, there is an urgent need to define and evaluate innovative and multi-target projects to reduce obesity risk behaviors and health inequalities and the present study aims to present the study protocol of FIVALIN a FBI that pretends to achieve this goal.

**Methods:**

We will conduct a quasi-experimental design within 60 Community Child Centers (CCC) in Barcelona metropolitan area. Each cluster (CCC) will be assigned by convenience to the intervention and control groups. For the whole study, a total of 810 children aged 812years and 600 parents will be recruited during 3 consecutive editions (1st 2019/2020; 2nd 2020/2021; 3rd 2021/2022) of 10months each. The action is a regular multicomponent health-promotion intervention targeting children, families, and CCC. All activities are based on the Motivational Interviewing (MI) approach and will focus on promoting good dietary habits, physical activity, appropriate screen time and sleep duration, and psychological well-being. The control group participate in a unique workshop on general awareness of healthy lifestyles for families. We will perform a comparative analysis of the evolution of weight status, healthy lifestyles, and socioeconomic variables, between the intervention and control groups.

**Discussion:**

There is a need for more evidence on how to target and evaluate holistic interventions in low SES families. Our multi-targeting intervention for obesity prevention tackles risky behaviors that go beyond diet and physical activity (PA). Therefore, future interventions can effectively promote all the behavioral domains that determine trends in the weight status.

**Trial registration:**

ISRCTN Registry: ISRCRN12682870. Registered 9 July 2020. Retrospectively registered.

Protocol version: 30 April 2021, version 5.

**Supplementary Information:**

The online version contains supplementary material available at 10.1186/s12887-021-02697-x.

## Background

Pediatric obesity is considered one of the most serious public health issues of the twenty-first century [1]. In Spain, there is a special concern due to the high prevalence of overweight and obesity affecting the 34.9% of children aged 816years [2]. This prevalence is one of the highest rates in Europe [3]. Overweight and obesity have several consequences, as almost every organ system can be severely damaged, leading to cardiovascular and musculoskeletal diseases, metabolic complications, gastrointestinal disorders, and pulmonary dysfunction [4]. In addition, in children and adolescents, these health conditions are related to psychosocial disorders, such as depression, anxiety, and low self-esteem [5, 6]. These disorders are caused by an interaction between individual and contextual factors [7]. There is a well-studied association between obesity and some personal determinants, such as dietary habits [8], physical activity (PA) [9], sleep [10, 11], and psychological well-being [12, 13]. Similarly, environmental variables, such as parental and socioeconomic characteristics, likely contribute to the problem [13,15]. Indeed, higher levels of childhood obesity have been consistently found in children with a low socio-economic status (SES), leading to health inequalities [16].

Inequalities in childhood obesity seem to have increased in developed countries during the last two decades [16, 17]. These imbalances are related to economic development, cultural factors, and social and health policies [18, 19]. In high-income countries, low SES is associated with 16% higher risk of overweight, and 43% higher risk of obesity in children aged 015years [20]. In Spain, the percentage of 2- to 17-year-old children with obesity is 11.45% higher in families where mothers have a lower SES [21]. In particular, in Catalonia, 0- to 14-year-old children of mothers with a low level of education have a 5.33% higher level of obesity, in comparison to children of highly educated mothers [22]. A recent study in Catalonia between 2006 and 2016 showed that this health condition tends to increase or be maintained in children from deprived areas, whereas richer regions showed a downward trend [23]. In particular, in Barcelona city, different factors play an important role on childhood obesity prevalence: social class, geographical origin, family status, and the district of residence [24].

The reasons why children from low SES are at higher risk of becoming overweight are likely to be multifaceted and determined by different parameters: i) structural factors, such as food advertising and marketing [25]; ii) community factors, like neighborhood [26] (demonstrated in the Spanish context) [27]; and iii) individual lifestyles factors, such as daily PA [28]. However, family environment also plays an important role in the development of obesity in children [14]. Family Based Interventions (FBI) demonstrated positive results in preventing childhood obesity, but SES contributes to the complexity of reaching positive results [14, 29]. More evidence is needed to better understand parents influence on school age childrens lifestyles from disadvantaged backgrounds, which evidence suggest a strong relationship from early childhood [30]. Moreover, only a limited number of FBIs-targeted risk behaviors other than diet and PA [31, 32]. The successful FBI that pretend to produce a positive effect on weight status and lifestyles should follow a multicomponent and multilevel approach [33,35]. This means that educational family interventions should also tackle political and social factors. Moreover, they should involve the key sectors where families spend their daily time such as school, primary care centers, sport organizations, leisure time facilities, etc. in order to build full community-based interventions.

The FIVALIN study protocol will use a multiple behavioral approach to simultaneously target diverse risk behaviors, such as healthy eating, PA, screen time, sleep quality and duration, and psychological wellbeing. In this way, our study will give a more comprehensive view of the value of FBIs in preventing childhood obesity among low SES families.

## Methods

### Study design

To determine the effects of the FIVALIN project, we will perform a quasi-experimental design with a control group.

### Aim

The aim of this manuscript is to describe the study protocol of the FIVALIN (Acronym of FItness, VAlues and Healthy LIfestyles) project. This is an FBI to prevent pediatric obesity in 8- to 12-year-old children, through promoting healthy lifestyles, among low-income families in Barcelona.

### Subjects

The study will involve low SES children aged 812years and their families from 60 Community Child Centers (CCC) in the metropolitan area of Barcelona. A CCC is a childcare center that promotes the education of children from low income families by providing them academic support and emotional education after school. A total of 810 children and 600 parents will be included in the intervention groups, and likewise for the control groups. These population samples will be distributed among three consecutive editions of the FIVALIN project (20192020, 20202021, and 20212022), and will include at least one adult and one child per low-income family. A total of 30 CCC (15 in the intervention group and 15 as controls) will be involved per edition. CCC from control group will be invited to be part of intervention group on the subsequent edition, and15 new CCC will be recruited in the control groups of the second and third editions. In total, after the three edition, 60 CCC will be enrolled in the project (see Table1). It is not expected that CCC participating in the intervention group during first or second edition will continue providing the intervention in subsequent editions. 18 socioeconomic vulnerable children (8- to 12-year-old) and their families will participate per CCC. Therefore, considering the intervention and control groups, we expect a total participation of 540 children and 400 adults per edition. Participants will be recruited by CCC, by inviting families that are already participating in their activities. They receive communicative material (flyers and leaflets) to introduce the project and motivate them to participate. For convenience, recruitment and allocation will be conducted by Gasol Foundation (GF), considering the characteristics of the CCC: activities, number of educators, parents involvement, etc.

**Table 1:** Number of CCC per FIVALIN edition

CCC from the	1st edition	2nd edition	3rd edition
Intervention group	1 to 15 CCC *(new)*	CCC from the control group of the 1 edition 16 to 30 CCC	CCC from the control group of the 2 edition 31 to 45 CCC
Control group	16 to 30 CCC *(new)*	31 to 45 CCC *(new)*	46 to 60 CCC *(new)*

### Inclusion and exclusion criteria for participants

#### Inclusion parameters

- Children aged 812years and their families from a low SES.
- Enrollment in a CCC.
- Informed consent signed by parents/legal representatives.

#### Exclusion parameters

- Children or parents/legal representative showing psychological or physical disadvantages that make it impossible to implement the assessment protocol and project activities.

### Sample size calculation

We estimate that it will be necessary to include 270 children and their parents, both in the intervention and control groups, per edition of the FIVALIN project. We expect a statistically significant decrease in body mass index (BMI) z-score of 0.15 in the intervention group, in comparison to the control group. From the PRE to POST evaluations, for each of the three editions. Previous community intervention [35] that aimed to prevent childhood obesity through the promotion of healthy lifestyles, and were carried out in the same geographical context and among children of the same ages. They showed a mean zBMI reduction of 0.10. The FIVALIN project is a more intense intervention and, for this reason, we expect a higher zBMI reduction.

We assume a 0.05 alpha risk, a 0.2 beta risk in a bilateral contrast, and 20% loss to follow-up. We expect to recruit significantly less families with 2 children in the age range of 8 to 12years, than families with one child. Therefore, we performed a simplified approximation of the sample size by counting only for one outcome (zBMI of one child). The sample size was calculated using the online sample size and power calculator GRANMO (IMIM-Spain).

### Intervention

The FIVALIN project is an FBI addressed to school age children (8- to 12-year-old) and their parents/caregivers. It has been created to be part of a Multi-Level Multi Component (MLMC) intervention that pretends to prevent childhood obesity from early ages, and along all the developmental stages, until adulthood. The pre-adolescence stage is a key period to sustain the healthy lifestyles promotion activities to prevent a deterioration at subsequent stages.

The FIVALIN project will be developed in three editions (1st 2019/2020; 2nd 2020/2021; 3rd - 2021/2022) of 10months each. The plan will be coordinated by the multidisciplinary health team of the GF, supported by educators of the CCC and a trained team of volunteers. We expect that the GF team will be stable. To ensure intervention consistency, we will activate different strategies (see Additionalfile1). Volunteers will be recruited by GF. They will contact the entity and, after a welcoming training, they will be involved in the project activities.

**Fig. 1:**
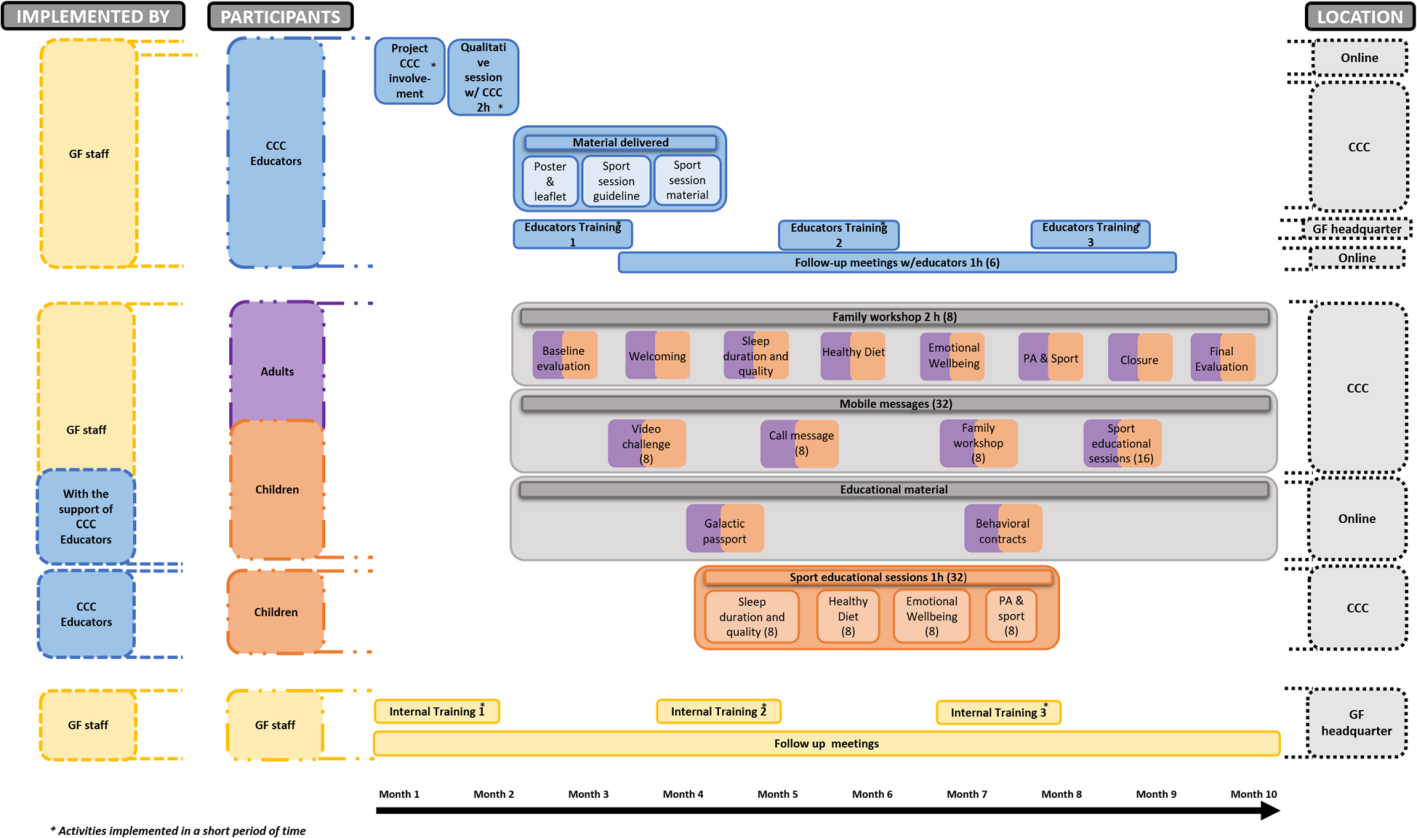
FIVALIN High intensity intervention group modality

**Fig. 2:**
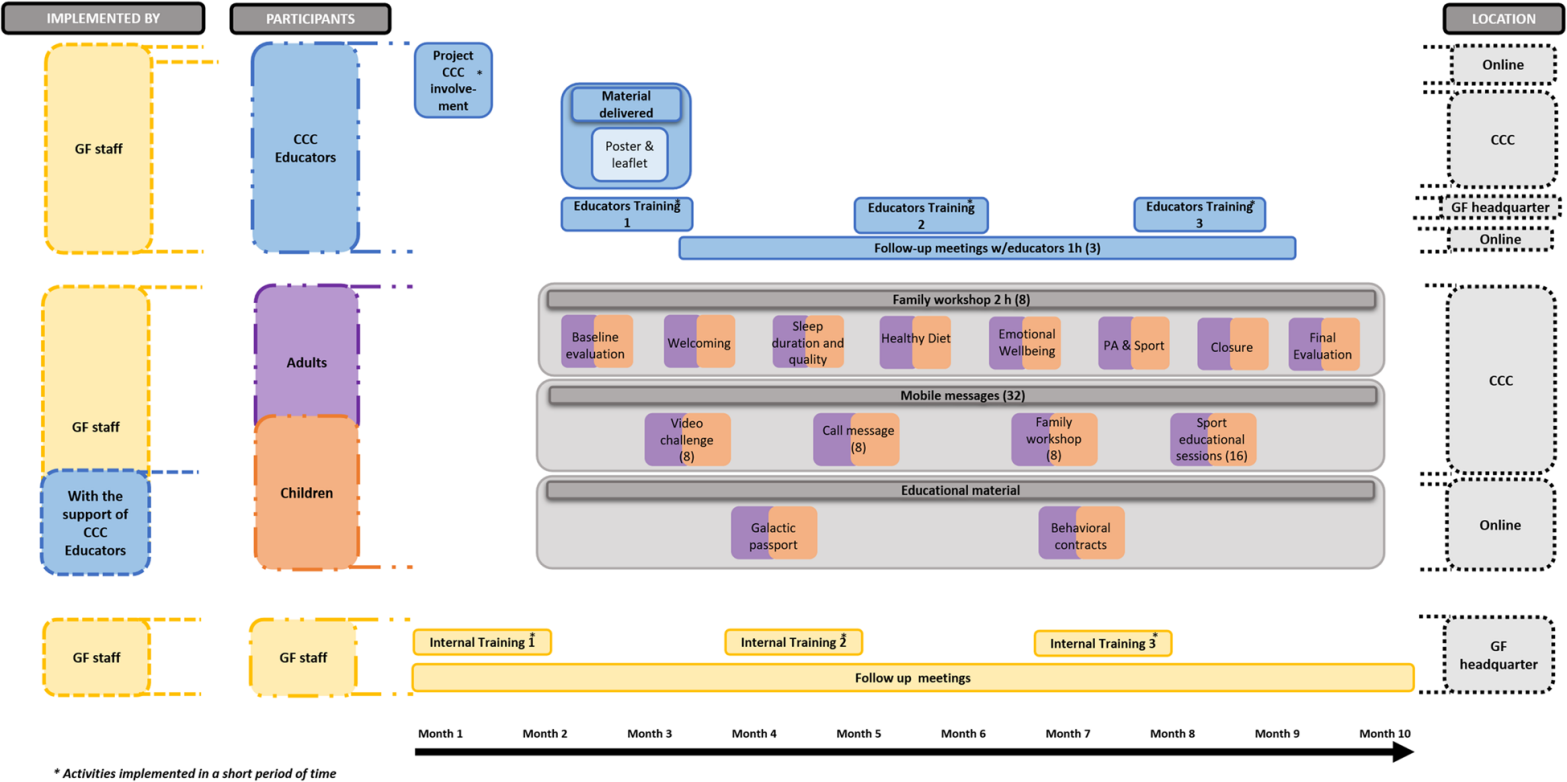
FIVALIN Low intensity intervention group modality

**Fig. 3:**
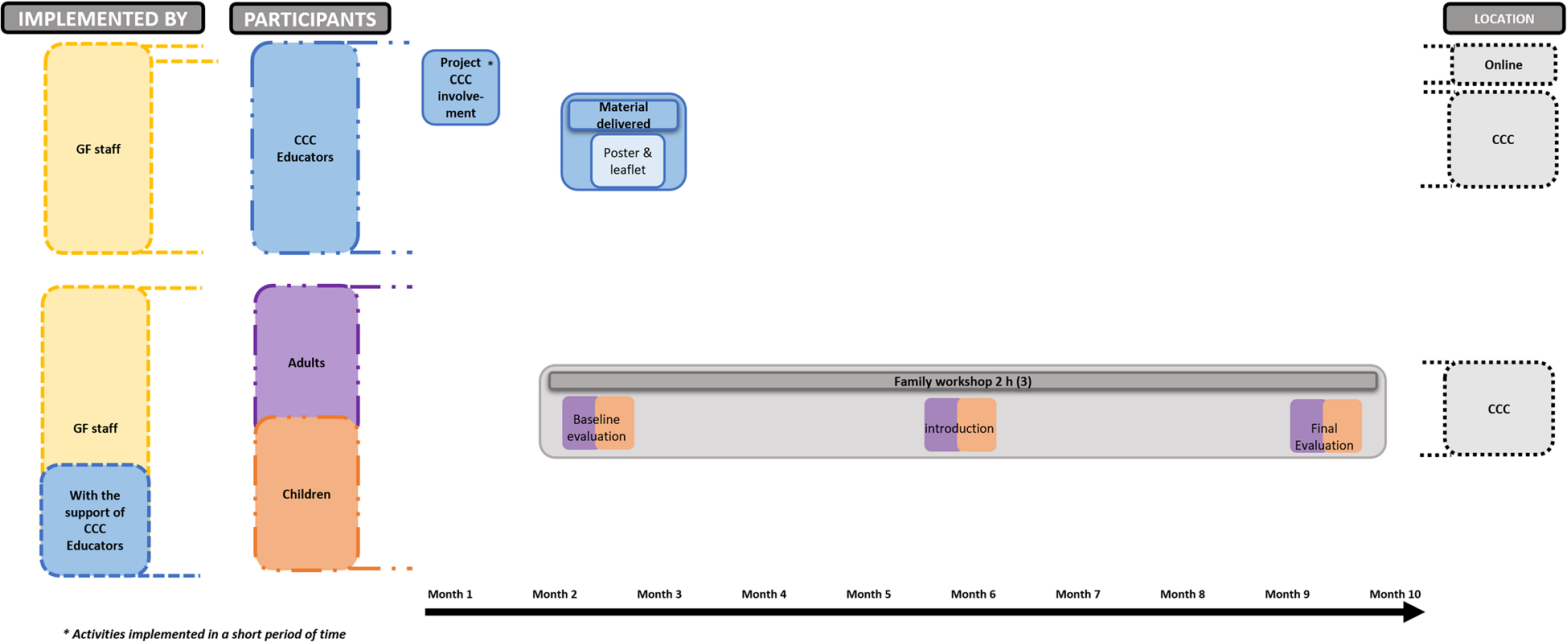
FIVALIN control group modality

The study will be conducted after school time in CCC in the metropolitan area of Barcelona. In CCC, parents are encouraged to be part of their childrens education. For the intervention group, we define two modalities of participation (low and high), depending on the project intensity. The main difference between the two modalities is that children participating in the high intensity modality will do an extra activity, attending 32 sports educational sessions implemented by CCC educators. Each CCC will choose the modality that best fits their internal organization (see Table2 and Figs.1, 2 and 3)*.* Additionally, educational material, mobile messages, and videos will be sent to families in order to reinforce the health behaviors promoted during the workshops and sport educational sessions. Moreover, mobile messages will remind workshops date and hour to promote the participants attendance. CCC participating in the control group will be invited to be part of the intervention group in the next edition. The control group will receive the usual care provided in the CCC program plus a pre-evaluation and post-evaluation session. Moreover, they will receive a family workshop aimed to generate healthy lifestyle awareness and engagement, in order to reduce shortcomings in the post-evaluation.

**Table 2:** FIVALIN project activities description by participation options

Activity description	Intervention		Control
	High intensity	Low Intensity	
**CCC**			
**Email and phone calls** to invite CCC to participate in the project.	Yes	Yes	Yes
**Communication material delivered**: 1 poster per CCC and 1 project introduction leaflet to each participant family.	Yes	Yes	No
**1 qualitative session implemented** with CCC educators (2h) before the project starts in the CCC to know participants family sociodemographic characteristics, their healthy lifestyles, and the CCC particularities.	Yes	Yes	No
**3 training (4h/training)**: face to face training with all the CCC participants educators every 2 months in the GF headquarter to promote the FIVALIN community, and share project methodology, theoretical basis, and project follow-up indicators.	Yes	Yes	No
**Sport educational sessions guide delivered to CCC**. The guide contains all the sport educational sessions that should be implemented by the CCC educators. For each healthy habit, the guide includes: i) Health messages definition; and ii) Sport educational sessions description: 8 sport educational sessions for health topic.	Yes	No	No
**Sport material box delivered to CCC** with all material needed to implement the sport educational sessions.	Yes	No	No
**Follow-up online meetings** (1h/meeting) with CCC educators.	Yes. 6 meetings	Yes. 3 meetings	No
**Children**			
**32 sport educational sessions** (1h/session) implemented by CCC educators using the sport educational sessions guide and sport material delivered. For each healthy habit, 4 health messages are promoted. For each healthy habit, 2 sport educational sessions are implemented.	Yes	No	No
**Family (children and adults)**			
**8 family workshops** (2h/workshop) involving a group of 15 to 20 children and parents from each CCC implemented in the CCC by GF staff with the support of CCC educators.	Yes. 8 Workshops: (1) Baseline evaluation (2) Welcoming (3) Sleep duration and quality (4) Healthy Diet (5) Emotional Wellbeing (6) PA & Sport (7) Closing (8) Final evaluation		Yes. 3 Workshops: (1) Baseline evaluative (2) General health awareness (3) Final evaluation
**32 mobile messaging** sent to participants families by using text, images, and infographics methods: (1) 8 mobiles messages, 1 before each family workshop, to recall families and promote their attendance. (2) 8 mobile messages, 1 after each family workshop, to reinforce the health topics promoted in the workshops. (3) 16 mobile messages, 1 after each cycle of 2 sport educational sessions dedicated to each healthy habit to reinforce it.	Yes	Yes	No
**8 challenges videos** done by high profile supporters (such as professional athletes and celebrities) posing a healthy challenge as a family related to one health topic.	Yes	Yes	No
**Health promotion educational material** shared with families after each workshop to reinforce the health messages shared in the sessions and workshops.	Yes	Yes	Yes
**GF staff**			
**Weekly meeting** (1h) with project coordinators.	Yes	Yes	Yes
**3 training** (4h/training): face to face training with all GF staff that coordinate and implement the project.	Yes	Yes	Yes
**Voice message report** after each family workshop.	Yes	Yes	Yes
**Ongoing support** with project coordinators to tackle all the project needs.	Yes	Yes	Yes

The project uses theoretical models based on social cognitive theory, such as: i) ASE (Attitude, Social influence, and Efficacy) model [36]; ii) Transtheoretical model [37]; and iii) Motivational interviewing (MI) [38]. Furthermore, the study will also use the Resiliency Theory [39] (see Additionalfile2). Finally, we will implement a multiple behavioral approach that considers dietary habits, PA and sport, screen time, sleep duration and quality, and psychological wellbeing, among children and their parents.

**Fig. 4:**
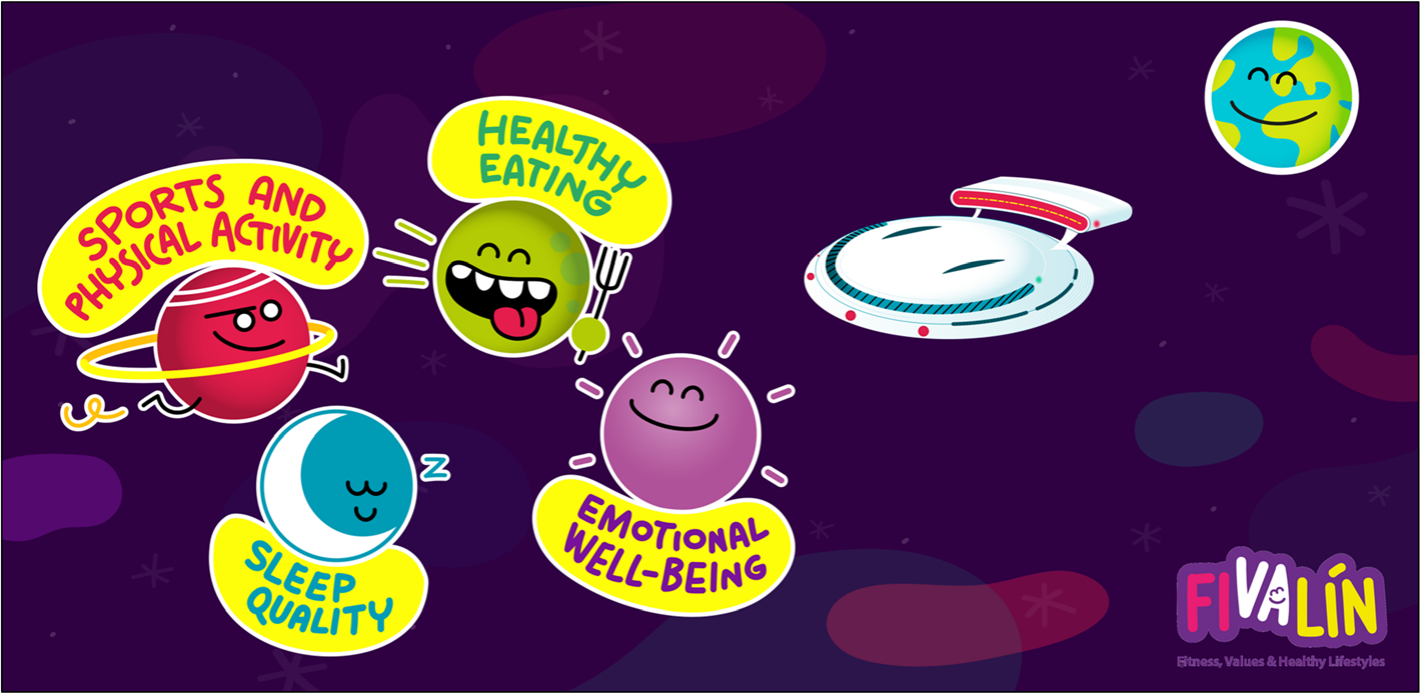
The Healthy Galaxy characters

**Fig. 5:**
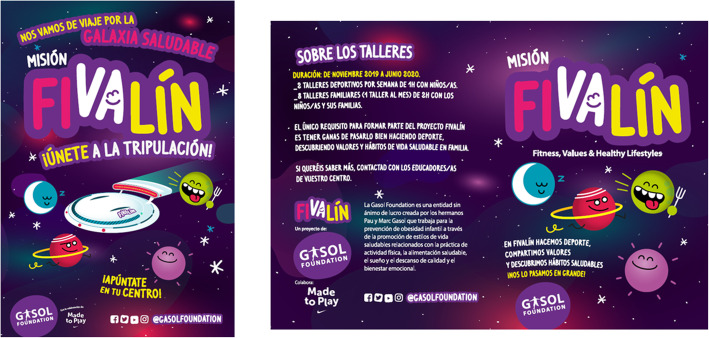
The Healthy Galaxy Communication material. Poster and Flyer example

**Fig. 6:**
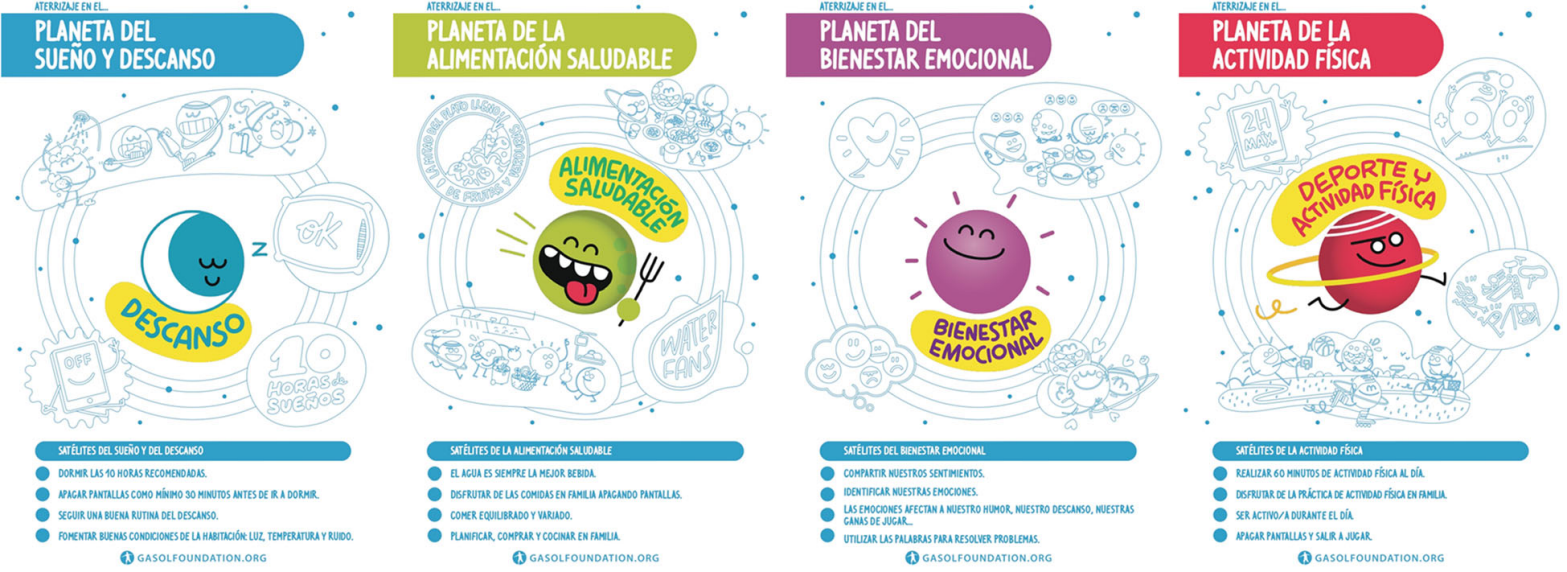
Educational material included in family activities

Healthy lifestyles will be implemented by using a pedagogical metaphor for the determinants of childhood obesity, *the Healthy Galaxy* [40]. The metaphor is based on a fantastic recent human story, where healthy habits decided to take off from planet Earth for a new Healthy Galaxy, where they created 4 planets: PA, healthy eating, sleep quality and duration, and emotional wellbeing (see Figs.4, 5 and 6). This story will motivate and promote families participation, while relating all the activities to one aim: families must return the healthy habits to the earth by travelling to the *Healthy Galaxy*. They will go around the galaxy on a spacecraft called FIVALIN and, back on the planet Earth, they will have another mission: spread the word about everything they learned.

**Fig. 7:**
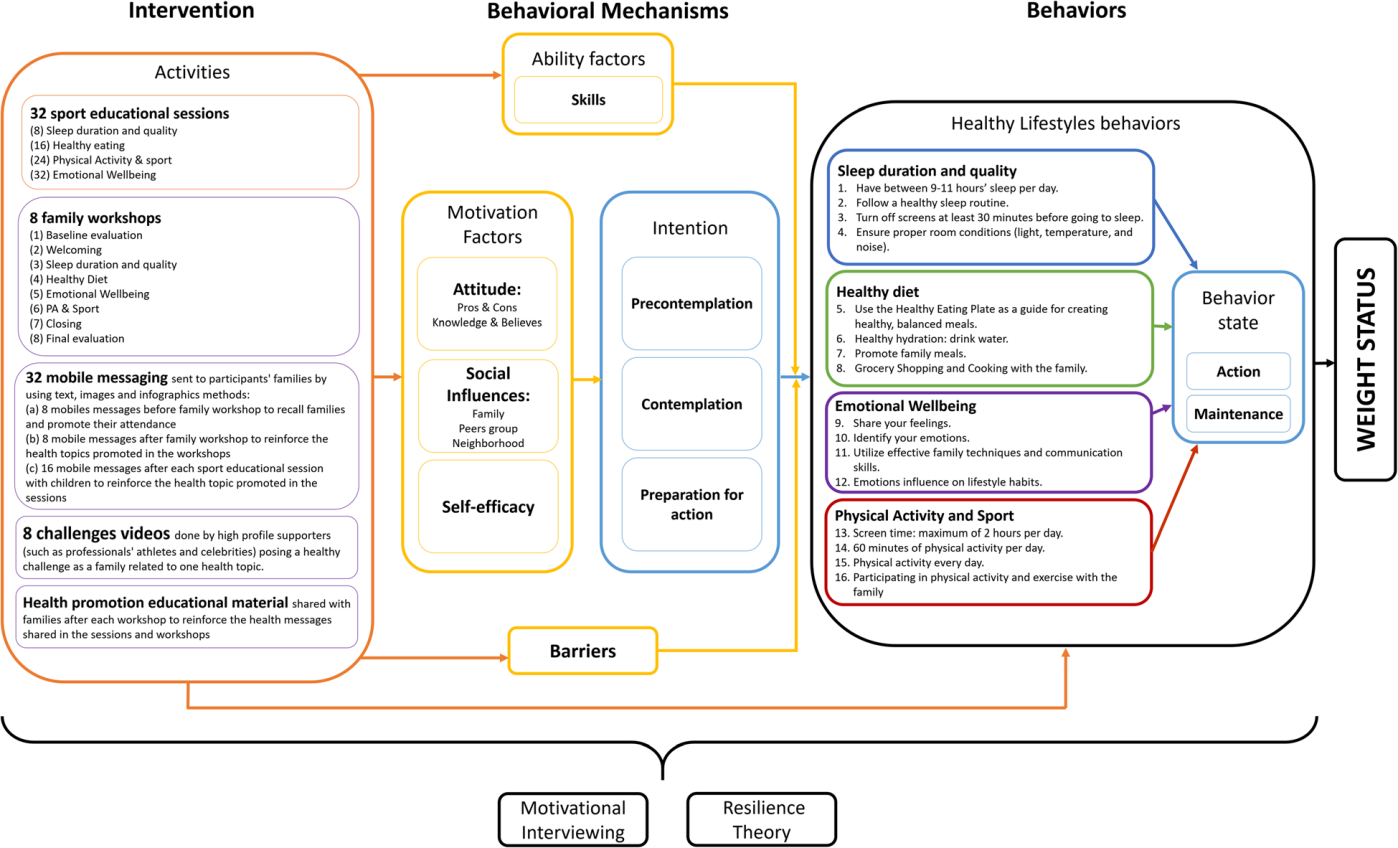
FIVALIN causal pathway

A FIVALIN causal pathway was created based on I-Change Model [41] (an integration of ASE model and Transtheoretical model) to clarify the logical framework that relates the intervention, behavioral mechanisms, behaviors, and weight status (*see* Fig.7).

The following strategies will be applied to promote adherence and prevent attrition during the project planification and implementation: i) carry out a qualitative session in each participant CCC and community before starting the intervention; ii) facilitate the logistics for attending the workshops by choosing CCC located in the families own communities and providing a childcare service for children under 5years; iii) implement participative and respectful activities during the family workshops, to highlight each positive lifestyle behavior, independently of the cultural background, helping to generates a positive family attachment; iv) deploy a process evaluation protocol to identify areas for improvement, considering families, educators, and CCC opinions; v) consider various individual strategies based on the Motivational Interviewing principles directly addressed to parents (making individual follow up calls, using incentives, sending mobile messages to recall and thanks their participation in the project family workshops).

In conclusion, the plan consists of a multicomponent intervention with strategies at different levels (childrens education, families, and centers organization), that include several activities.

### Data collection

#### Anthropometric variables

Weight will be recorded to the nearest 100g using an electronic scale (SECA 899), and height will be recorded to the nearest 1mm (without shoes) using a portable SECA 213 stadiometer. Waist circumference will be assessed in the narrowest zone between the lowest rib and iliac crest, in the vertical position, with a flexible non-stretch tape measure (SECA 201). Every device is systematically calibrated and all measurements will be performed by a GF-trained researcher.

#### Online questionnaires

All lifestyle data will be reported using online questionnaires. Parents will report information on screen time, diet, sleep duration and quality, and psychological wellbeing of their children. They will also take a questionnaire on their own lifestyles and sociodemographic characteristics. Children will complete a self-assessment questionnaire on PA and physical condition, with the help of GF researchers and CCC educators.

#### The online questionnaires

A. **Data collection for Children**

1. Diet Quality (KIDMED questionnaire)
2. Physical Activity (Physical Activity Unit- 7 Items - PAU-7S)
3. Physical Condition (The international Fitness Scale - IFIS)
4. Screen time-based and Sedentary Behavior (Screen time-based sedentary behaviour questionnaire - SSBQ)
5. Sleep Hours (Sleep-Habits Survey for Adolescents questionnaire - SHSA)
6. Sleep Quality (Bedtime Issues, Excessive Daytime Sleepiness, Night Awakenings, Regularity and Duration of Sleep and Snoring questionnaire - BEARS)
7. Behavior (Strengths and Difficulties Questionnaire - SDQ)

##### Diet quality

Diet quality will be evaluated using the KIDMED index [42], which is based on 16 items with a dichotomous (yes/no) response format, reported by parents. The KIDMED test was created to estimate adherence to the Mediterranean diet in children and young adults. It is based on principles that are aligned or not with the Mediterranean dietary pattern. The index ranges from 4 to 12 and is based on a 16-question test. Answers indicating a negative or positive behavior with respect to the Mediterranean diet are assigned a value of 1 or+1, respectively. The sums of these values are classified into three levels: i)>8, optimal Mediterranean diet; ii) 47, improvement needed to adjust intake to Mediterranean patterns; iii)<3, poor diet quality.

##### Physical activity

Level of PA will be evaluated by using the Physical Activity Unified-7 item Screener (PAU-7S) (see Additionalfile3), which is self-reported by the child (validity results sent for publication and currently under review). The PAU-7S consists of 7 questions referring to the week before the test. Questions 13 address different types of PA, such as walking, team sports, and individual activity, respectively. Questions 46 address the context where the PA was practiced: schoolyard, after school and on weekends, and physical education classes, respectively. The last question refers to whether the child was ill during the previous week or was unable to perform PA for some reason. Questions 16 will be answered according to 5 possible options that refer to time spent (no activity=0min, <30min, 3060min, 6090min, and>90min). The questionnaire will give the total amount of minutes dedicated to PA during the week before the test.

##### Physical condition

Childrens physical condition will be assessed using the International Fitness Scale (IFIS) self-rating questionnaire [43]. IFIS is composed of five Likert-scale questions on childrens shape, in comparison to that of their friends. Children will evaluate their own overall physical condition, cardiorespiratory fitness (CRF), muscular fitness (MF), speedagility (SPAG), and flexibility. They will rank themselves as very poor, poor, average, good or very good. The appraisal of a good/very good overall physical condition, CRF, or SPAG indicates a healthy cardiovascular profile.

##### Screen time and sedentary behavior

Sedentary behaviors will be assessed using the Screen-Time-based Sedentary Behavior Questionnaire (SSBQ) [44], which is reported by parents. 6 questions inquire about behaviors such as: i) watching TV; ii) playing computer games; iii) playing console (video) games; iv) using internet for non-study reasons (hobbies); v) using for study reasons; and vi) studying (outside the school schedule). Parents will separately answer the 6 questions for weekdays and weekends, and will indicate the usual time devoted to the 6 habits: 0min; <30min; 3060min; 60120min; 120180min; 180240min; and>240min. The number of sedentary minutes per day will be rated according to the following categories: 1=0min, 2=15min, 3=45min, 4=90min, 5=150min, 6=210min, and 7=241min, respectively. Weekly sedentary time is calculated by taking the mean time in the selected category and applying this formula: [(weekdays*5)+(weekend days*2)]/7. The total sedentary score is obtained by summing the time reported in each category. Higher scores indicate higher levels of screen time and sedentary behavior.

##### Sleep duration and quality

Sleep patterns will be assessed using the BEARS Questionnaire [45], which will be completed by parents to evaluate potential sleep problems in children. We will consider 5 major sleep domains, such as: B=Bedtime issues; E=Excessive daytime sleepiness; A=night Awakenings; R=Regularity and duration of sleep; and S=Snoring. Parents will give a yes or no response for each domain.

Parents will also complete a Sleep-Habits Survey for Adolescents (SHSA) [46], which will serve to estimate childrens sleep duration. 4 questions will inquire about usual sleeping and waking behaviors. Parents will report the time their children go to bed and wake up during school days and at weekends.

##### Psychological wellbeing

For behavioral screening, the Strengths and Difficulties Questionnaire (SDQ) [47], a brief questionnaire for 3- to 16-year-old children, will be completed by parents. The SDQ collects data on 25 attributes: 5 scales including 5 items each. The scales consist in: i) emotional symptoms; ii) conduct problems; iii) hyperactivity/ inattention; iv) peer relationship issues; and v) prosocial behavior. Parents have three possible choices for each item on these scales: Not True, Somewhat True, and Certainly True. For all scales, higher scores indicate more problems, except for the last scale, where higher scores correspond to fewer difficulties in prosocial behavior. In addition, children will answer a brief self-report question on their own health.

##### Other data collected

We will collect data on birth weight and breastfeeding using a standard questionnaire to be completed by the adults.

B. **Data collection for Parents**

1. Diet Quality (short Diet Quality Screener - sDQS)
2. Physical Activity (REGICOR Short Physical Activity Questionnaire)
3. Sleep Duration
4. Perceived Stress (Perceived Stress Scale - PSS)

##### Diet quality

Diet quality will be analyzed by using a self-report questionnaire: the short Diet Quality Screener (sDQS) [48]. Parents will be asked to base their responses on their dietary behaviors over the previous 12months, reporting their usual intake of 18 food items, grouped in 3 categories. These categories are based on recommended frequencies of food intake. Consumption frequency will be rated 1, 2, or 3 for healthy food items, and 3, 2, 1 for the unhealthy food items. All scores will be added up. Outcomes will range from 18 (low quality diet) to 54 (high quality diet).

##### Physical activity

PA will be assessed by using a self-report REGICOR [49] questionnaire on exercise type, frequency (days per month), and duration (minutes per day). The questionnaire also provides categorical information about PA at work or in everyday life and gives a weekly total amount of training minutes.

##### Sleep duration

Parents sleep duration will be calculated by asking about their usual sleeping and waking times on weekdays and weekends.

##### Perceived stress

The Perceived Stress Scale (PSS) [47] will be used to measure the perception of stress. This self-report questionnaire includes several direct queries about current levels of stress experienced. Questions refer to feelings and thoughts during the previous month. The complete version of the test contains 14 questions with 5 response categories each (0=never, 1=almost never, 2=occasionally, 3=often, 4=very often). PSS scores are obtained by reversing responses (e.g., 0=4, 1=3, 2=2, 3=1, and 4=0) to 4 positively stated questions (4, 5, 7, and 8), and then summing the values. Scores of 013, 1426, and 2740 indicate low, moderate, and high perceived stress, respectively.

##### Other data collected

Additional data will be collected about the following items:

- Socioeconomic status (annual income, and educational and occupational levels)
- Smoking status
- Family meals per week
- Family PA per week
- Family postal address
- Number of years parents and children have been living in Spain
- Household characteristics

C. **Data collection for educators and CCC**

We will collect data on CCC characteristics, such as: (i) location; (ii) total number and ages of children, and family workshop and sport educational sessions attendance; (iii) number of people of the staff; and (iv) if applicable, number and characteristics of health promotion actions implemented in the CCC. The latter will be collected at the beginning of the project. Moreover, we will also collect CCC staff data on the following parameters: (i) socio demographic characteristics; (ii) educational level and studies; and (iii) grade of motivation and awareness through healthy lifestyle promotion, at the beginning and at the end of each edition.

### Evaluation plan

We will collect all data for each edition at baseline and 10months after the intervention (post-evaluation). Moreover, a follow up evaluation will be performed in the intervention group 12months after the post-evaluation, to study whether the impact of FIVALIN is sustained in the long term. After the assessment sessions, each participating family from the intervention and control groups will receive an incentive (e.g., a reusable water bottle, a technical shirt, or a ball).

The intervention fidelity and process evaluation indicators will be assessed in different ways, such as: i) periodical project coordinators meetings; ii) follow-up meetings and evaluation sessions with CCC educators; iii) voice audios after project activities recorded by GF educators; iv) registration of family attendance and level of satisfaction after each workshop; and v) a qualitative assessment at the end of each project edition (focus groups and interviews to key stakeholders). The evidence derived from the previous assessment activities will be used with 2 aims, such as: (i) improve the project management after each edition; and (ii) incorporate future improvements to the project basis, once the three editions will be implemented and the final data of the present study will be gathered. The project methodology, activities, assessment tools, staff profile, and the expected outcome will be the same for the 3 editions because a final evaluation of pooled data will be performed.

### Statistics

We will analyze the pooled data from the three timely different editions with a 10-month intervention each. Data clean-up will be performed to minimize errors. To compare groups, we will use the Student-t test for continuous variables and chi-square test for categorical variables. Allocation to the intervention group will be performed at the CCC level (clusters) by convenience. Therefore, generalized estimating equation (GEE) models will be fitted to assess intervention effect on BMI z-score and other secondary outcomes at individual level. To assess the outcome at family level, we will apply mixed models.

Due to the small number of clusters, GEE models estimation will be followed by t test with the Kauermann and Carrol-corrected sandwich estimator. In alternative, we will use the Wald t test with the FG-corrected sandwich estimator, depending on the variation in cluster size (function saws from R package saws) [50]. FG-corrected sandwich estimation is not possible with mixed models. For this reason, we will apply GEE models for the analyses. BMI z-score and other secondary outcomes at baseline will be used as covariates to adjust the models. Final models will be adjusted for baseline covariates such as: age, sex, mothers educational level, adherence to the Mediterranean diet, PA, and the corresponding anthropometric variable (function mgee from R-packagesaws). A fidelity score will also be included as a covariate in the models. Differences between the intervention and control group were considered significant if *p*<0.05. All analyses will be carried out using SPSS V21.0 and R.

## Discussion

The epidemic of overweight and obesity presents a major worldwide challenge for chronic disease avoidance and health throughout life. Preventing overweight in children may have the greatest long-term effects [51]. Pediatric obesity is more common in population groups with a lower SES [20]; therefore, strategies and programs need to prioritize the inclusion of vulnerable groups [52]. The FIVALIN project is an FBI that uses a novel approach to prevent overweight among children through promoting healthy lifestyles. The plan targets low-SES families and is implemented via CCC.

A major strength of this project is that we will be able to carry out a follow-up 12months after the post-evaluation. Another strength is the multiple behavioral approach. A systematic review revealed gaps in the behavioral domains targeted in childhood obesity prevention, with only 16% of the analyzed intervention directed at four behavioral domains (PA, Nutrition, Media use, and Sleep) [31]. However, it is important to target and evaluate all the behavioral domains that influence weight status. Indeed, FBI studies measuring secondary outcomes have found significant changes in two or more behavioral aspects [29].

Although the internal features of the CCC prevent us from conducting a properly randomized trial, the socio-demographic characteristics of the intervention and control groups of the first edition of FIVALIN are similar. It could be argued that, since lifestyle variables will be measured by self-administered questionnaires, the participants` low level of education may influence their ability to understand and answer them. Nevertheless, families with the lowest reading and comprehension skills will receive the support of trained researchers to complete the questionnaires, and biases are expected to be similar in the intervention and control group.

One limitation of the FIVALIN project could be the self-selection biases. Considering that participation is voluntary, parents with concerns about their childrens weight or health behavior might be more interested in the study. Moreover, CCC educators individual awareness and motivation could influence the effects of the intervention. The project also poses the challenge of how to engage parents. Parents involvement has to be considered as a potential agent of change in the development of their childrens health-related behaviors [14]. A systematic review indicated that the level of parental involvement appeared to positively impact the effectiveness of interventions on childrens weight and energy balance-related behaviours [53]. Another compilation report suggests that interventions promoting participant engagement may be more beneficial to disadvantaged groups than to higher literacy/SES status families [54]. Various parental characteristics are associated with low participation: low SES, single parenthood, difficult living circumstances, stress, family dysfunction, and belonging to a minority group ethnicity [55]. Therefore, it is important to take these risk factors into account in order to improve the efficacy of preventive actions [15]. We will undertake several strategies to minimize the effects of the above-mentioned barriers as described in the methods section. The reliance on self-report over objectively measured is a weakness commonly assumed by the intervention studies. Another limitation is not having a complete fidelity assessment to analyze project adherence and competence beyond family attendance and few actions related to process assessment. Although the fidelity plan must be improved, we have prioritized the definition and implementation of a feasible assessment plan to not produce an overburden to families and CCC educators. Thus, in the FIVALIN project, we take the opportunity to implement assessment methodologies as voice message, that could contribute to future intervention addressed to families experimenting vulnerability, to establish a feasible strategy to monitor fidelity.

Finally, the potential bias based on non-randomized CCC group intervention assignment would be also a limitation because, the CCC and educators could have a higher level of motivation to participate in the high intensity modality. As highlighted, this could influence project effectiveness. Data regarding CCC and staff characteristics will be collected and are described in methods section.

In conclusion, the FIVALIN project will contribute to prevent childhood obesity among socioeconomically vulnerable families. It will do so by using a multiple behavioral approach to simultaneously target healthy eating, PA, screen time, sleep quality and duration, and psychological wellbeing. Moreover, the behaviors targeted are considered to be at the same level of importance and will be analyzed as both primary and secondary outcomes. This approach will provide evidence on the influence of each behavioral area on the evolution of the weight status [29].

## Supplementary Information

**Additional file 1.** Strategies to train new staff and assure consistency of the intervention. List of strategies to ensure intervention consistency and train new staff.

**Additional file 2.** Theoretical models applied in the FIVALIN project by project activities. List of project activities where Theoretical models are applied.

**Additional file 3.** Physical Activity Unified-7 item Screener (PAU-7S). English version of Physical Activity Unified 7 items Screener (PAU-7S) administered to children.

**Additional file 4.** INFORMATION SHEET AND INFORMED CONSENT FIVALIN STUDY.

## Data Availability

The datasets used and/or analyzed during the current study will be available from the corresponding author on reasonable request.

## Abbreviations

FIVALIN: Fitness, Values, and healthy Lifestyles
SES: Socioeconomic Status
FBI: Family Based Interventions
CCC: Community Child Centers
GF: Gasol Foundation
PA: Physical Activity
BMI: Body Mass Index
ASE: Attitude, Social influence, and Efficacy
MI: Motivational Interviewing
PAU-7S: Physical Activity Unified-7 item Screener
IFIS: International Fitness Scale
CRF: Cardiorespiratory Fitness
MF: Muscular Fitness
SPAG: SpeedAgility
SSBQ: Screen-Time-based Sedentary Behavior Questionnaire
BEARS: Bedtime issues; Excessive daytime sleepiness; night Awakenings; Regularity and duration of sleep; Snoring
SHSA: Sleep-Habits Survey for Adolescents
SDQ: Strengths and Difficulties Questionnaire
sDQS: short Diet Quality Screener
PSS: Perceived Stress Scale
CEIm: Ethics committee
