# Supplementary material for: Family-based intervention to prevent childhood obesity among school-age children of low socioeconomic status: study protocol of the FIVALIN project
Source: BMC Pediatr. 2021 May 21;21:246. doi: 10.1186/s12887-021-02697-x (PMC8139065; doi:10.1186/s12887-021-02697-x)
Supplement: Supplementary file 1 — Additional file 1. Strategies to train new staff and assure consistency of the intervention. List of strategies to ensure intervention consistency and train new staff. [file 12887_2021_2697_MOESM1_ESM.docx]

**Additional file 1: Strategies to train new staff and assure consistency of the intervention:**

- All project staff have very similar socio-educational characteristics.
- A total of 3 internal trainings will be conducted during each project edition to assure an homogeneous implementation.
- A welcoming training is conducted by the project coordinators with the new staff as a complementary training.
- New staff are receiving personal support from the project coordinators during the first weeks till they are ready to implement the project activities.
- New staff participate as an extra staff on the project activities during the first weeks to see how the project implementation works.
- Weekly follow-up project coordinators (1h) are conducted to ensure a correct project development.
- After each project activity related to face-to-face family and CCC educators a voice message report is shared with all staff members. Project coordinators gives feed-back of each report to assure the consistency in the implementation procedures between CCC.
- Throughout the whole intervention different communication channels are used to be in contact with all staff members: online platforms, e-mail and voice messages.

CCC = Community Child Center
