# Supplementary material for: Family-based intervention to prevent childhood obesity among school-age children of low socioeconomic status: study protocol of the FIVALIN project
Source: BMC Pediatr. 2021 May 21;21:246. doi: 10.1186/s12887-021-02697-x (PMC8139065; doi:10.1186/s12887-021-02697-x)
Supplement: Supplementary file 2 — Additional file 2. Theoretical models applied in the FIVALIN project by project activities. List of project activities where Theoretical models are applied. [file 12887_2021_2697_MOESM2_ESM.docx]

**Additional file 2: Theoretical models applied in the FIVALIN project by project activities.**

| **Project activities** | **Theoretical models** | | | |
| --- | --- | --- | --- | --- |
|  | **Attitude, Social influence, and Self-efficacy – ASE model** | **Transtheoretical model** | **Motivational interviewing** | **Resilience Theory** |
| **Project CCC and involvement of the families:**   - Communication skills to introduce the project to CCC representatives are based on the principles of the Motivational Interviewing approach (express empathy, develop discrepancy, avoid arguments, adjust resistance, and support self-efficacy). - Contents of the communication material (poster and leaflet) are based on the principles of the Motivational Interviewing. |  |  | **x** |  |
| **Internal training to GF staff** **on the theory:**   - Theoretical basis training. - How to implement the communication of the theoretical basis of the project activities to family and CCC educators. | **x** | **x** | **x** | **x** |
| **Qualitative sessions:**   - Identification of the stage of change in which most participants' families are. These analyses help the educators and GF staff to provide appropriate health messages depending on the most predominant stage of change of each participant group of families (precontemplation, contemplation, preparation, action, maintenance, and termination). - Use of the principles of the Motivational Interviewing to communicate with educators and conduct the qualitative session. |  | **x** | **x** |  |
| **Educational material**:   - Educational material content is defined based on the psychosocial determinants (attitude, social influence, and self-efficacy) and stages of change. - A behavioral contract related to each healthy topic is delivered to each participant family. This behavioral contract: (i) reinforces healthy habits related to the different psychosocial determinants; (ii) promote the deployment of the 40 Developmental Assets® defined by Search Institute, within families. These assets consist of preventative measures, positive experiences, and qualities that children need to grow up healthy, caring, and responsible; (iii) promote the interiorization of the Motivational Interviewing principles within families. | **x** | **x** | **x** | **x** |
| **CCC educators training**:   - Theoretical basis training. - How to implement theoretical basis of project activities and family communication. | **x** | **x** | **x** | **x** |
| **Family workshops & sport educational sessions**:   - Family workshops & sport educational sessions are defined considering the psychosocial determinants of health behaviors. - Dynamic contents for adults’ workshops are defined by following the stages of change. - The communication of the CCC educators and the GF staff with the families is based on the Motivational Interviewing principles. - During family workshops and sport educational sessions, the 40 Developmental Assets® are promoted. | **x** | **x** | **x** | **x** |
| **Mobile messages**:   - Mobile messages content and video messages are addressed to reinforce one of the psychosocial determinants and also considering one of the six stages of change. - Communication style is based on the Motivational Interviewing principles. | **x** |  | **x** |  |
