# Supplementary material for: Family-based intervention to prevent childhood obesity among school-age children of low socioeconomic status: study protocol of the FIVALIN project
Source: BMC Pediatr. 2021 May 21;21:246. doi: 10.1186/s12887-021-02697-x (PMC8139065; doi:10.1186/s12887-021-02697-x)
Supplement: Supplementary file 3 — Additional file 3. Physical Activity Unified-7 item Screener (PAU-7S). English version of Physical Activity Unified 7 items Screener (PAU-7S) administered to children. [file 12887_2021_2697_MOESM3_ESM.docx]

**Additional file 3:** Physical Activity Unified - 7 item Screener (PAU-7S)

**Physical Activity Questionnaire**

We want to know what your level of physical activity was. This includes sports, running, dancing, walking, jumping, and any activity that involves movement.

- There is no right or wrong answers.
- Answer the questions sincerely.

Think about the last week, the last 7 days. Notes with a cross "x" your answer for each corresponding question: walking, playtime, free time to physical education, team sports, and individual sports. Leave blank if you have not done any activity listed below.

|  | | **Monday** | **Tuesday** | **Wednesday** | **Thursday** | **Friday** | **Saturday** | **Sunday** |
| --- | --- | --- | --- | --- | --- | --- | --- | --- |
| **How long have you played team sports?** | 0 minutes |  |  |  |  |  |  |  |
|  | Less than 30 minutes |  |  |  |  |  |  |  |
|  | 30 minutes-1 hour |  |  |  |  |  |  |  |
|  | 1 hour-1 hour and 30 minutes |  | **X** |  | **X** |  |  |  |
|  | More than 1 hour 30 minutes |  |  |  |  |  | **X** |  |

**For example: Paul is an 8 year old boy playing basketball. He plays every Tuesday and Thursday for 1 hour-1 hour and a half. Also, on Saturday morning, he played longer than 1 hour and a half. Therefore, the answer to the question on team sports would be:**

**Now think about your last week, the last 7 days. Note with a cross "X" your answer to each question. Leave it if you have not done any activity listed below:**

1) Which days of the week do you walk (to go to school in the morning, afternoon after school, weekends, walking tour)? Think of any walking. How long did you walk each day more or less?

|  | | **Monday** | **Tuesday** | **Wednesday** | **Thursday** | **Friday** | **Saturday** | **Sunday** |
| --- | --- | --- | --- | --- | --- | --- | --- | --- |
| **How long have you been walking?** | 0 minutes |  |  |  |  |  |  |  |
|  | Less than 30 minutes |  |  |  |  |  |  |  |
|  | 30 minutes-1 hour |  |  |  |  |  |  |  |
|  | 1 hour-1 hour and 30 minutes |  |  |  |  |  |  |  |
|  | More than 1 hour 30 minutes |  |  |  |  |  |  |  |

2) At recess time at school (break time), which days have you played games involving movement, running, or jumping (sports, play catch together, jumping, rope)? And how long each day (considering morning and afternoon break times)?

|  | | **Monday** | **Tuesday** | **Wednesday** | **Thursday** | **Friday** |
| --- | --- | --- | --- | --- | --- | --- |
| **How long have you played movement games?** | 0 minutes |  |  |  |  |  |
|  | Less than 30 minutes |  |  |  |  |  |
|  | 30 minutes-1 hour |  |  |  |  |  |
|  | 1 hour-1 hour and 30 minutes |  |  |  |  |  |
|  | More than 1 hour 30 minutes |  |  |  |  |  |

3) After school or during the weekend (in your free time), which days did you play games that involved movement, running, or jumping (play catch together, rope, bicycle, rollerblading)? And, more or less, how long each day?

|  | | **Monday** | **Tuesday** | **Wednesday** | **Thursday** | **Friday** | **Saturday** | **Sunday** |
| --- | --- | --- | --- | --- | --- | --- | --- | --- |
| **How long have you played movement games?** | 0 minutes |  |  |  |  |  |  |  |
|  | Less than 30 minutes |  |  |  |  |  |  |  |
|  | 30 minutes-1 hour |  |  |  |  |  |  |  |
|  | 1 hour-1 hour and 30 minutes |  |  |  |  |  |  |  |
|  | More than 1 hour 30 minutes |  |  |  |  |  |  |  |

4) Which days have you attended physical education classes at school?

|  | **Monday** | **Tuesday** | **Wednesday** | **Thursday** | **Friday** |
| --- | --- | --- | --- | --- | --- |
| **Which days did you do physical education?** |  |  |  |  |  |

5) Which days have you played team sports in a club or as an extracurricular sport (Football, Basketball, Handball, Hockey, Water polo)? Think of the training and matches. And, more or less, how long each day?

|  | | **Monday** | **Tuesday** | **Wednesday** | **Thursday** | **Friday** | **Saturday** | **Sunday** |
| --- | --- | --- | --- | --- | --- | --- | --- | --- |
| **How long have you played team sports?** | 0 minutes |  |  |  |  |  |  |  |
|  | Less than 30 minutes |  |  |  |  |  |  |  |
|  | 30 minutes-1 hour |  |  |  |  |  |  |  |
|  | 1 hour-1 hour and 30 minutes |  |  |  |  |  |  |  |
|  | More than 1 hour 30 minutes |  |  |  |  |  |  |  |

6) Which days did you play individual sports in a club or as an extracurricular sport (Athletics, rhythmic gymnastics, dance-ballet, Tennis, judo-Karate-Taekwondo, skating, swimming)? Think of training and competitions. And more or less, how long each day?

|  | | **Monday** | **Tuesday** | **Wednesday** | **Thursday** | **Friday** | **Saturday** | **Sunday** |
| --- | --- | --- | --- | --- | --- | --- | --- | --- |
| **How long have you played team sports?** | 0 minutes |  |  |  |  |  |  |  |
|  | Less than 30 minutes |  |  |  |  |  |  |  |
|  | 30 minutes-1 hour |  |  |  |  |  |  |  |
|  | 1 hour-1 hour and 30 minutes |  |  |  |  |  |  |  |
|  | More than 1 hour 30 minutes |  |  |  |  |  |  |  |

|  | | **Monday** | **Tuesday** | **Wednesday** | **Thursday** | **Friday** | **Saturday** | **Sunday** |
| --- | --- | --- | --- | --- | --- | --- | --- | --- |
| **How long have you played individual sports?** | 0 minutes |  |  |  |  |  |  |  |
|  | Less than 30 minutes |  |  |  |  |  |  |  |
|  | 30 minutes-1 hour |  |  |  |  |  |  |  |
|  | 1 hour-1 hour and 30 minutes |  |  |  |  |  |  |  |
|  | More than 1 hour 30 minutes |  |  |  |  |  |  |  |

7) This last week, were you sick (cold, leg pain, etc.) or was there something that would not let you do the activities that you normally do?

| Yes |  |
| --- | --- |
| No |  |

What happened? _______________________________________________

**THANK YOU FOR ANSWERING THE QUESTIONS ON PHYSICAL ACTIVITY!**

**OR ANSWERING THE QUESTIONS OF PHYSICAL ACTIVITY!**
