# Supplementary material for: Family-based intervention to prevent childhood obesity among school-age children of low socioeconomic status: study protocol of the FIVALIN project
Source: BMC Pediatr. 2021 May 21;21:246. doi: 10.1186/s12887-021-02697-x (PMC8139065; doi:10.1186/s12887-021-02697-x)
Supplement: Supplementary file 4 — Additional file 4. INFORMATION SHEET AND INFORMED CONSENT FIVALIN STUDY. [file 12887_2021_2697_MOESM4_ESM.docx]

**Appendix:**

**INFORMATION SHEET AND INFORMED CONSENT FIVALIN STUDY**

We ask you to read this information sheet and informed consent and ask any questions before agreeing to participate in the study.

**Description of the research and your participation**

With this document, we request you and your child the participation consent in the study associated with the FIVALIN project coordinated by the Gasol Foundation. The purpose is to promote an improvement in the level of physical activity, lifestyles, and weight status of children and adults participating.

The FIVALIN project offers two participation options: (A) Intervention group, that will carry out the project actions: 8 family workshops and 32 sports educational sessions with children; (B) Control group, which will carry out a family workshop during the 2019-2020/2020-2021/2021-2022 academic year. The participating centers will be assigned to group A or group B, offering the control group the possibility to participate as an intervention group during the following academic year 2020-2021/2021-2022. Both groups will carry out a first evaluation during the months of November-December 2019/2020/2021, and a second evaluation between May and June 2020/2021/2022. The participants (parents/legal representatives, and their children) will be invited to answer questionnaires about their lifestyle: hours of physical activity, physical condition, hours of screen, sleep and rest, food, and emotional well-being. In turn, anthropometric measurements will be carried out by a health professional in an intimate space and individually. For measurements, participants must remove their shoes and wear a shirt and pants. To measure the waist circumference, it will be necessary to lift the shirt to the level of the navel. For measurements, participants must remove their shoes and wear a shirt and pants. To measure the waist circumference, it will be necessary to lift the shirt to the level of the navel. For measurements, participants must remove their shoes and wear a shirt and pants. To measure the waist circumference, it will be necessary to lift the shirt to the level of the navel. Both children and adult referents will answer the questionnaires through a very intuitive and easy-to-use online platform and always in the presence of professionals from the Gasol Foundation who will guide them.

**Risks**

There are no risks associated with this research or derived from the tests to be carried out.

**Potential benefits**

The study will generate valuable information to know the impact of the FIVALIN project on the lifestyles and general health status of the participating population.

**Protection of confidentiality and data**

The files and databases generated by the study will be kept strictly confidential. The physical files will be archived under lock and key and with limited access to the study coordinating staff, and the electronic information will be encrypted and stored under a confidential password protected file folder. In the files and databases in which confidential information or health data are available, the name or personal data of the participants will not appear. The Gasol Foundation's program team will identify the participants by means of a numerical code during the sessions. Once the data has been encrypted, they will only be accessible by research personnel linked to the Gasol Foundation. The study follows the guidelines of the Helsinki declaration on ethical principles in research. Your personal data will be processed in accordance with Regulation (EU) 2016/679 of April 27^th^, 2016, and Royal Decree 1720/2007, of 21 December, which approves the Regulation implementing Organic Law 15/1999, of 13 December, on the Protection of Personal Data. The latter approves the Regulations for the development of Organic Act 3/2018 of 5 December, on the Protection of Personal Data and the guarantee of digital rights and Law 14/2007 on Biomedical Research. For this, we inform you that: (1) The purpose of data collection and processing is the management of the study; (2) The legitimation is the consent of the interested parties to carry out the activities described; (3) The data will not be kept for longer than necessary for the activity for which they were collected, unless there is a legal obligation; (4) The data will not be communicated to other third parties, except for legal obligation. You have the right to withdraw consent, to exercise the right of access, rectification, portability, and deletion of your data and the limitation or opposition to its treatment, by sending a letter to the GASOL FOUNDATION, or by email to proteccion.datos@gasolfoundation.org. Likewise, you have the right to file a claim with the Spanish Agency for Data Protection in the event of non-compliance by the entity. As established in the current regulations of the Law of the Information Society and Electronic Commerce, we request your consent to send information from the FIVALIN study, via email or equivalent electronic means of communication. The interested party is informed that they may revoke their consent to send communications at any time by sending an email to protección.datos@gasolfoundation.org.

The results obtained from this study can be published in scientific journals anonymized and the identity of the participants will never and under no circumstances be provided.

**Voluntary participation** Your participation in this research is voluntary. You can choose not to participate and withdraw your informed consent at any time. In this case, your data will be removed from the database. In no case can you be penalized, even if you decide not to participate or withdraw from the study.

**Contact information** If you have any questions, please contact the Gasol Foundation Program Manager and Principal Investigator, Dr. Santiago Felipe Gómez at +34 93 834 66 00 or somez@gasolfoundation.org.

**Consent**

*(Location) ......................................................................., (day/month/year) ………. / ………. /……….*

**Me,** *(name and surname of the father / mother / legal representative):*

………………………………………………………..................................................... **as** *(father/mother/legal representative*) .............................................. **of the child or adolescent (***Name and surname)* …………………………………………….……………………………………………………………………………………., from the center ……………………………………………………………., born on *(day/month/year) ………. / ………. /……….* Gender:  Male  Female

Email address …………………………………………………………………………………..……………………… *(for the questionnaires).*

Contact mobile phone: …………………………………………………………………………………………………………… *(for the questionnaires)*

I have read this informed consent sheet and have had the opportunity to ask my questions. Check the box x that corresponds to your decision, **fill in the data and sign the document.**

** I DO NOT** give my consent to participate in this study and therefore I sign below.

Reason: ..........................................................................................................................................

Firm: ………………………..……………...

** YES,** I give my consent to participate in this study and therefore I sign below and mark and complete the data in box A, B, or C, that correspond to my situation.

1. **** I specify the name, surname, and signature of the two parents or legal representatives:

Name of father / mother or legal representative 1: ………………………………………………

Firm: ………………………

Name father / mother or legal representative 2: ………………………………………………

Firm: ………………………

1. **** I confirm that the other parent does not object to our child's participation in the study.

Firm: ………………………

1. **** I confirm that the signer is the sole legal guardian.

Firm: ………………………

Principal investigator and signature: Dr. Santiago Felipe Gómez Santos.
